# Exclusive breastfeeding changes in Brazil attributable to pacifier use

**DOI:** 10.1371/journal.pone.0208261

**Published:** 2018-12-19

**Authors:** Gabriela Buccini, Rafael Pérez-Escamilla, Maria Helena D’Aquino Benicio, Elsa Regina Justo Giugliani, Sonia Isoyama Venancio

**Affiliations:** 1 Departament of Nutrition, Universidade de São Paulo, São Paulo, São Paulo, Brazil; 2 Departament of Social and Behavioral Sciences, Yale School of Public Health, New Haven, Connecticut, United States of America; 3 Department of Pediatrics, Universidade Federal do Rio Grande do Sul, Porto Alegre, Brazil; 4 Department of Director, Instituto de Saúde, São Paulo, São Paulo, Brazil; TNO, NETHERLANDS

## Abstract

**Background:**

Identifying key interventions to increase exclusive breastfeeding duration has been a challenge. Pacifier use has been associated with exclusive breastfeeding discontinuation in Brazil. However, the proportion of the improvement in exclusive breastfeeding duration attributable to pacifier use remains unknown.

**Research aim:**

Quantify the proportion of increases in exclusive breastfeeding prevalence that can be attributed to reduced pacifier use over time.

**Methods:**

Secondary data analyses of two nationally representative cross-sectional surveys conducted in States’ capitals in 1999 and in 2008 (N = 42,395 Brazilian infants under 6 months of age). We estimated the fraction of exclusive breastfeeding prevalence improvements that could be attributed to pacifier use based on multilevel regression analysis.

**Results:**

From 1999 to 2008, there was an increase of 15.2 percentage points in exclusive breastfeeding prevalence and a decrease of approximately 17 percentage points in the prevalence of pacifier use among infants under 6 months. Reduction in pacifier use explained an increase in 5.5 percentage points’ exclusive breastfeeding rates. If pacifier use were to decrease from 41.6% (prevalence in 2008) to 14% (as found in New Zealand), there would be an expected additional increase in exclusive breastfeeding of approximately 12 percentage points.

**Conclusions:**

About one-third of the improvements in EBF prevalence observed in Brazil over a decade can be attributed to the corresponding decline in pacifier use.

## Introduction

Evidence of both short- and long-term benefits of breastfeeding on infant survival, health, and development, as well as on maternal health and human capital, are well documented [1, 2]. Over the last two decades, the prevalence of exclusive breastfeeding (EBF) in infants under 6 months increased worldwide, from 24.9% in 1993 to 35.7% in 2013 [1]. In an effort to further improve EBF prevalence, the 2012 World Health Assembly (WHA) endorsed breastfeeding as one of the key global nutrition targets to foster a healthy, equitable, and sustainable future for individuals and nations. This target specifically calls for increasing the prevalence of EBF among infants up to six months of age to at least 50% by 2025. In Brazil EBF prevalence has increased over time, from 3.1% in 1986 to 41.3% in 2008, however at the current rate it would take another 6 years to reach the EBF rate of 50% [3]. This is an optimistic estimate as a recent study found that the rate of improvements in EBF prevalence has recently slowed down in Brazil. Indeed, for the first time in decades the EBF prevalence has not increased within the last seven years (2006–2013) [4]. Therefore, the EBF WHA goal will not be met unless key modifiable risk factors are identified.

Pacifier use may be a common modifiable risk factor for EBF discontinuation [5–7]. However, systematic reviews and meta-analyses investigating the relationship between pacifier use and shorter EBF duration have shown divergent results [8, 9]. Unfortunately the only two randomized clinical trials [10, 11] available to find out if there is a causal relationship between pacifier use and EBF duration have major methodological shortcomings that limit both the internal validity and the generalizability of findings [8]. Cross-sectional studies assessing the same population at different time points [7, 12] have consistently demonstrated a negative association between pacifier use and EBF duration, and 20 longitudinal studies point to a dose-response relationship [8].

Recommendations for pacifier use vary worldwide [13]. The American Academy of Pediatrics recommends the use of pacifier to prevent Sudden Infant Death Syndrome (SIDS) and that pacifiers can be introduced after breastfeeding is well established, at approximately 3 to 4 weeks of age [13, 14]. By contrast, WHO used to discourage the use of pacifiers in breastfed children as one of the “Ten Steps to Successful Breastfeeding” upon which the Baby-Friendly Hospital Initiative (BFHI) has been based [15]. Recently, the 2018 revised BFHI guidelines revised this step to counsel mothers on the use and risks of pacifiers. Clearly this is not a straight forward recommendation as it needs to take into account the potential benefits (e.g., SIDS) and risks (e.g. interference with EBF).

In this context, we undertook this study to estimate the potential impact of reducing pacifier use on EBF in Brazil. This study has important implications for Brazil and the rest of the world as to our knowledge it is the first time that the proposed attributable risk estimation is conducted anywhere in the world.

## Materials and methods

This study used data from the First and Second National Surveys of Breastfeeding Prevalence conducted in 1999 and 2008, respectively, in all urban areas of Brazilian state capitals and in the Federal District. The surveys investigated feeding practices of infants under 1 year of age attending national immunization campaigns. These surveys have been found to provide precise population estimates, given the wide coverage of these campaigns [16].

Parents provided informed verbal consent to participate in the survey and all data were fully anonymized before being accessed for this study. The study protocol was approved by the Research Ethics Committee of Faculdade de Saúde Pública, Universidade de São Paulo, Brazil (protocol n^o^ 766158, approved July 18 2014).

### Sampling and data collection

The procedures employed at the two survey waves related to sampling and data collection were very similar and are described in detail elsewhere [17]. In brief, participants were selected based on two-stage complex sampling procedures. In the first stage, considering the number of children immunized in each center, random drawing of the immunization center within the capital immunization centers were selected; in the second stage, infants waiting to be vaccinated were also systematic randomly selected. Mothers were interviewed using a standard questionnaire with closed ended questions. Data were collected on socio-economic and demographic infant, maternal and household characteristics, pacifier use, and consumption of breast milk and other foods by the infant in the previous 24 hours [17, 18].

One capital was excluded from the analysis because it did not participate in the 1999 survey. Moreover, only infants under 6 months were included, as the primary outcome was EBF. The analytical sample was comprised by 42,395 participants (n = 24,810 infants from the 1999 survey and 17,585 from the 2008 survey).

### Data analysis

#### Temporal variations in the prevalence of EBF

As recommended by WHO, infants were considered to be exclusively breastfed if they received only breast milk (i.e., not any other solid or liquid foods) in the last 24 hours [18]. This information was obtained by asking mothers about breast milk consumption as well as tea, juice, water, and other milks, in addition to questions on the intake of solids in the last 24 hours. All these questions had had a dichotomous answer (yes/no). Temporal variations in EBF prevalence were estimated based on EBF prevalence in 1999 and 2008.

The outcome variable was EBF discontinuation, i.e., not being exclusively breastfed in the last 24 hours before the interview.

#### Variables

The main independent variable was pacifier use in the last 24 hours (yes/no). Statistical analyses controlled for the following covariates that had previously been found to predict EBF [2, 19]: infant sex (female/male), type of delivery (vaginal/cesarean), maternal age (<20 years/20-34 years/≥35 years), maternal education level (0–8 years/9-12 years/>12 years), and maternal work status (does not work outside home/is on maternity leave or works outside home); infant age (0–60 days/61-120 days/121-180 days). Capital of residence was included as a contextual variable in the statistical models.

#### Multilevel analysis

Analysis of the influence of pacifier use and covariates on temporal variations in EBF prevalence was performed in four stages. In the first stage, factors that could have influenced temporal variations in EBF were assessed. At first, changes in the results obtained for pacifier use and covariates were described, comparing results obtained in 1999 and 2008. Variables showing similar distributions in the two surveys (p>0.05) were not included in the next stage.

**Fig 1:**
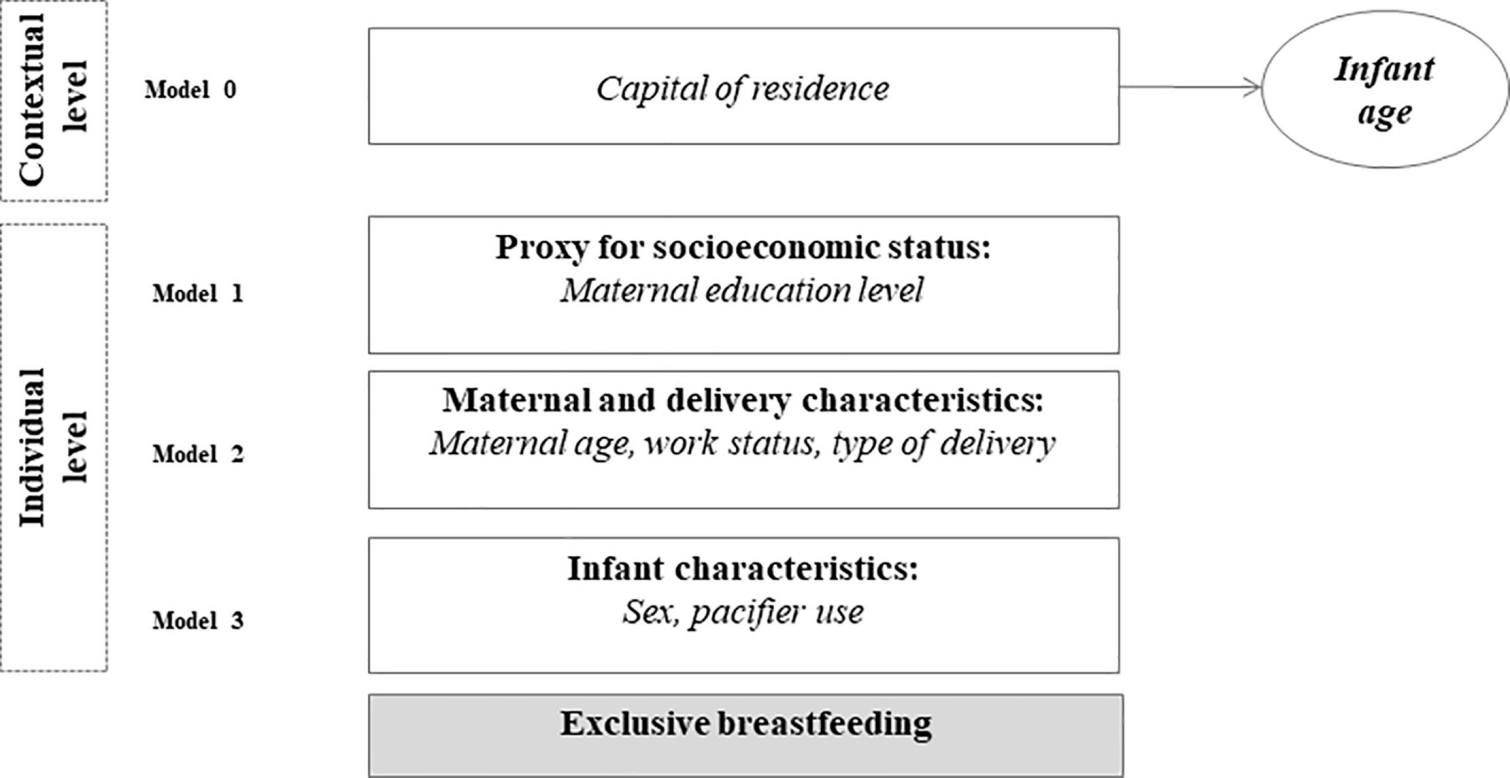
Statistical modelling steps for determining the influence of pacifier use on exclusive breastfeeding discontinuation in Brazilian state capitals and the Federal District, according to hierarchical blocks, Brazil, 1999 and 2008.

In the second stage, associations between EBF discontinuation and the factors studied in 1999 and 2008 were assessed using Poisson multilevel analysis with robust variance adjusted for infant age. At this stage, state capitals comprised level 2 (contextual variable) and individual maternal and infant data comprised level 1 (individual variables). In order to estimate the individual effect of the variables on the outcome, a hierarchical approach was used [20], following a statistical modelling steps established *a priori* (Fig 1) [2, 19]. The variable capital of residence was added to the initial model (model 0), organizing individual data according to the clusters used in the multilevel analysis. Subsequently, in model 1, the first variable included was maternal education (proxy for socioeconomic status); this variable was adjusted for in subsequent models. Likewise, variables related to maternal and delivery characteristics were added in model 2 to model 1 variables, and were controlled for in the subsequent model. A similar procedure was adopted for variables related to infant characteristics (model 3). Significant variables were maintained in the model even if they became non-significant after the inclusion of following model for fitness of the model. The fitness of model adjustment was assessed using the 2-log likelihood test. A significance level of 5% was adopted for considering a factor associated to the outcome after adjustment for variables within the same model and models added before. In the multilevel analysis, the fixed effects/random intercept model described by Snijders & Bosker was used [21].

The third stage involved decomposition of the combined effect of different factors associated with temporal variation in EBF prevalence into components attributable to the individual evolution of each factor that remained associated with the outcome in the second stage of analysis. This effect was estimated by calculating generalized potential impact fractions (PIF) [22–24]. This estimate is an extension of the concept of population attributable fraction to situations in which the distribution of risk factors changes, but the factors are not necessarily eliminated [22–24]. This approach can be used to predict an increase in EBF prevalence, for example, as a result of decreasing, but not eliminating, pacifier use. According to this approach, it is possible to assume that exposure to the risk factor is relevant to the occurrence of the negative outcome and that decreasing this exposure would help to reduce the risk of experiencing the outcome [24].

In order to estimate the PIF of each factor associated with temporal changes in the prevalence of EBF discontinuation, the equation proposed by Kleinbaum was used [23]:

$$\hat{\mathrm{PIF}}=\frac{\sum_{i=0}^{k}(P_{1999}–P_{2008})x\;I\hat{D}R}{\sum_{i=0}^{k}P_{1999}(I\hat{D}R)}$$

where P1999 and P2008 are the estimated proportions of the exposure factor at the 1999 and 2008 surveys, respectively; and $I\hat{D}R$ is the mean adjusted prevalence ratio of the exposure factor obtained from the 1999 and 2008 surveys. PIF were calculated considering the adjusted estimators obtained from the hierarchical models of the 1999 and 2008 surveys in the multilevel analysis, as well as the initial and final magnitude range of each factor assessed, given the complexity of the sample design.

This way, PIF allows to estimate the effect that would be expected from a change in the distribution of each of the factors assessed on EBF prevalence. However, the effects estimated for each determinant should not be summed, as they tend to add up to more than the combined effect of all factors for simultaneous changes in the five determinants. This limitation, which prevents the perfect decomposition of the combined effect of changes in the five variables, results from the hypothetical assumption that changes in each variable will precede changes in the other variables, which is not always the case. Still, the estimate obtained for each variable will indicate its relative importance in the evolution of the prevalence of EBF discontinuation in the period assessed [25]. Therefore, PIF can be properly interpreted as the expected proportional decrease in the prevalence of EBF discontinuation resulting from changes observed in the distribution of the exposure factors over the 1999–2008 period, i.e., the proportion of EBF discontinuation that could be avoided by decreasing the occurrence of the risk factors (e.g., pacifier use) in the population assessed [23].

Finally, the fourth stage of analysis the etiological fraction or population attributable fraction (FAP) was calculated by comparing the expected proportional decrease in EBF discontinuation calculated by PIF and the expected decrease resulting from elimination of the exposure factor [22–24].

#### Simulation of scenarios for different prevalence rates of pacifier use

Considering the prevalence of pacifier use in other countries, as reported in a multicenter study [26], we simulated four scenarios. For each of those scenarios, we estimated hypothetical PIF by investigating the association between decrease in pacifier use and EBF prevalence. In order to do that, we considered the 2008 survey as baseline for the simulation of possible “future” scenarios, and used the distribution of pacifier use and prevalence ratio found for the 2008 survey as reference for other calculations. Calculated this way, the hypothetical PIF represents the additional decrease in EBF discontinuation rates that could be achieved by decreasing the prevalence of pacifier use to the stipulated levels in the simulation.

The statistical analyses were performed with Stata software version 14.1, applying individual weighting factors of each survey to the standard error of the estimates. PIF was calculated using Excel version 14.1.0.

## Results

EBF rates increased 15.2 percentage points between 1999 and 2008, corresponding to a mean annual increase of 1.6%. There was also a significant decrease in pacifier use, of approximately 17 percentage points (Table 1).

**Table 1:** Prevalence of exclusive breastfeeding (%) and distribution of infants under 6 months of age and their mothers according to pacifier use and covariates in the First and Second National Surveys of Breastfeeding Prevalence in Brazilian state capitals; Brazil, 1999–2008.

	1999^a^ (n = 24,810)		2008^b^ (n = 17,585)		p^c^
	**N**	**%**	**N**	**%**	
Exclusive breastfeeding					<0.001
Yes	6,626	25.1	6,860	40.3	
No	18,184	74.9	9,816	59.7	
Pacifier use					<0.001
No	11,281	41.5	10,811	58.4	
Yes	13,133	58.5	6,496	41.6	
Infant age					<0.001
0–60 days	7,151	28.5	5,756	32.5	
61–120 days	8,579	34.4	6,032	34.1	
121–180 days	9,080	37.1	5,797	33.4	
Sex					0.65
Female	12,332	50.3	8,721	50.0	
Male	12,302	49.7	8,864	50.0	
Type of delivery					<0.001
Vaginal/forceps	15,481	61.5	8,805	51.5	
Cesarean	9,168	34.5	8,561	48.5	
Maternal age (years)					<0.001
≥20	18,956	79.0	12,850	83.2	
<20	5,590	21.0	2,890	16.8	
Maternal education (years of schooling)					<0.001
0–8	14,363	59.5	6,041	38.1	
9–12	7,885	33.0	7,519	48.5	
>12	1,778	7.4	2,245	13.4	
Maternal work status					<0.001
Does not work outside home/on maternity leave	18,106	72.1	12,744	84.1	
Works outside home	6,356	27.9	2,200	15.9	

^a,b^ Adjusted for sample weight of each municipality in each survey wave

^c^ *p*-value comparing 1999 and 2008 survey participants.

Pacifier use showed the highest prevalence ratio for EBF discontinuation at the two surveys, with a stronger magnitude of association in 2008 (Table 2). Lower maternal education, being a younger mother and mother working outside home were also associated with EBF discontinuation at the two waves. Cesarean, in turn, was a risk factor for EBF discontinuation only in the latest survey (Table 2).

**Table 2:** Poisson multilevel regression used to estimate the prevalence ratio of exclusive breastfeeding discontinuation according to maternal and infant characteristics adjusted for infant age in the First and Second National Surveys of Breastfeeding Prevalence in Brazilian state capitals^a^; Brazil, 1999–2008.

	Model 0^b^	Model 1^c^	Model 2^d^	Model 3^e^	
***First survey*, *1999***					
***Fixed effects*** Constant	0.58 (0.52–0.65)*	0.56 (0.50–0.64)*	0.55 (0.49–0.63)*	0.51 (0.44–0.60)*	
Maternal education (years of schooling)					
>12	-	**1**	-		-
9–12	-	**0.99 (0.94–1.03)**	-		-
0–8	-	1.04 (1.00–1.09)**	-		-
Maternal age (years)					
≥20	-	-	**1**		-
<20	-	-	1.09 (1.06–1.13)*		-
Type of delivery					
Vaginal/forceps	-	-	**1**		-
Cesarean	-	-	**1.02 (1.00–1.04)**		-
Maternal work status	-	-			
Does not work outside home/on maternity leave	-	-	**1**		-
Works outside home	-	-	1.04 (1.01–1.07)**		-
Pacifier use					
No	-	-	-		**1**
Yes	-	-	-		1.25 (1.15–1.34)*
***Random effect*** Capital—constant	0.014 (0.009–0.022)	0.014 (0.009–0.021)	0.014 (0.009–0.021)		0.013 (0.008–0.020)
***-2loglikelihood***	569367.52	552966.98	535563.52		524844.66
***Second survey*, *2008***					
***Fixed effects*** Constant	0.42 (0.38–0.46)*	0.38 (0.35–0.41)*	0.34 (0.31–0.37)*		0.29 (0.27–0.32)*
Maternal education (years of schooling)					
>12	-	**1**	-		-
9–12	-	1.05 (1.01–1.10)**	-		-
0–8	-	1.16 (1.11–1.22)*	-		-
Maternal age (years)					
≥20	-	-	**1**		-
<20	-	-	1.16 (1.13–1.19)*		-
Type of delivery					
Vaginal/forceps	-	-	**1**		-
Cesarean	-	-	1.03 (1.01–1.04)**		-
Maternal work status	-	-			
Does not work outside home/on maternity leave	-	-	**1**		-
Works outside home	-	-	1.21 (1.17–1.25)*		-
Pacifier use					
No	-	-	-		**1**
Yes	-	-	-		1.52 (1.44–1.59)*
***Random effect*** Capital—constant	0.012 (0.006–0.021)	0.013 (0.007–0.023)	0.012 (0.007–0.022)		0.012 (0.007–0.021)
***-2loglikelihood***	524923.26	463266.62	426540.00		417184.48

^a^ Individual data adjusted for sample weight of each municipality in each survey wave.

^b^ Model 0: capital of residence and infant age

^c^ Model 1: model 0 + maternal education

^d^ Model 2: model 1 + maternal age, type of delivery, maternal work status

^e^ Model 3: model 2 + pacifier use

* p<0.001

** p<0.05

**Fig 2:**
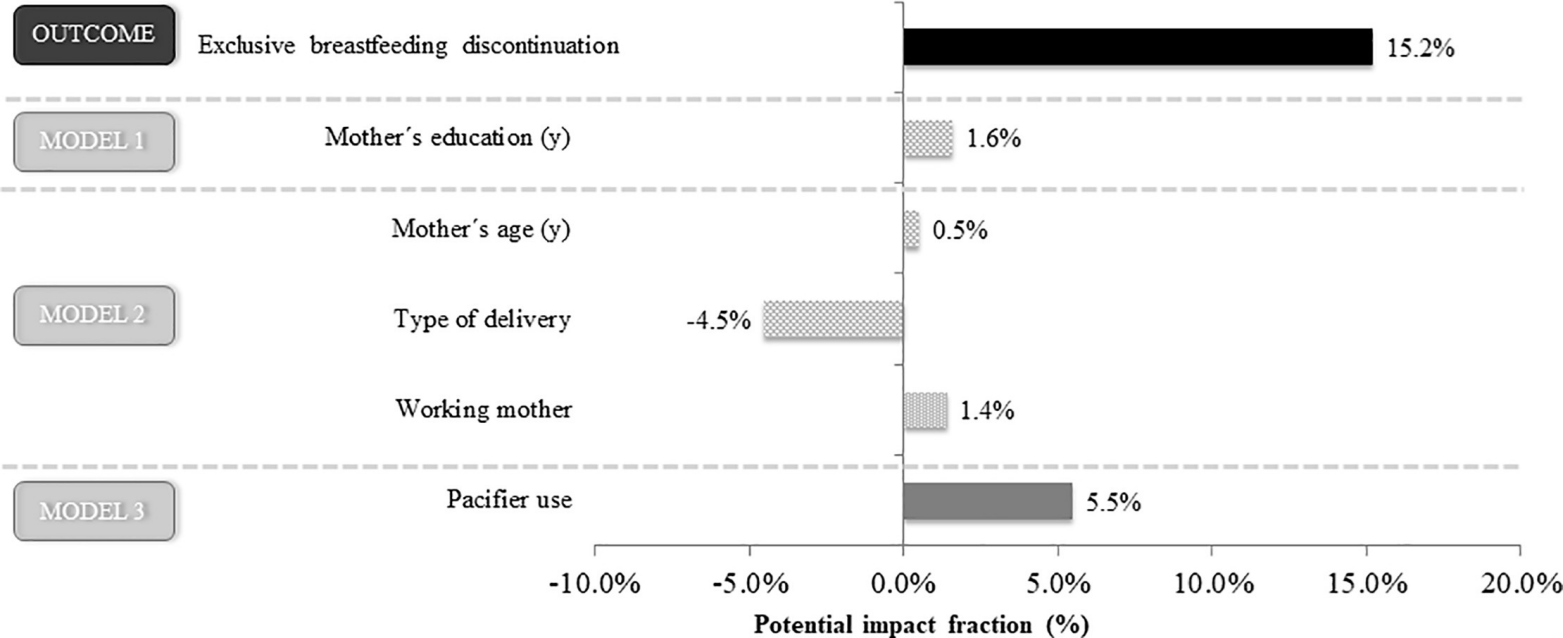
Relative participation (%) in the decrease of exclusive breastfeeding discontinuation in the period 1999–2008: The role of pacifier use.

Changes in the prevalence of pacifier use yielded a proportional decrease (PIF) of 5.5 percentage points in the prevalence of EBF discontinuation (Table 3 and Fig 2). Higher maternal education and age as well as access to maternity leave for the 6 months or not working outside also contributed to a proportional decrease (PIF) in the prevalence of EBF discontinuation (Table 3 and Fig 2).

**Table 3:** Expected decrease (%) in exclusive breastfeeding discontinuation according to temporal variations of pacifier use and covariates in the First and Second National Surveys of Breastfeeding Prevalence in Brazilian state capitals; Brazil, 1999–2008.

		*% 1999*^*a*^ *(n = 24*,*810)*	*% 2008*^a^ *(n = 17*,*585)*	*Prevalence ratio* *1999+2008*^b^	*% Proportional decrease* *1999–2008 (PIF)*^c^	*% Decrease eliminating exposure* *1999–2008 (FAP)*^d^
*MODEL1*	***Maternal education (years of schooling)***					
	*>12*	*7*.*4*	*13*.*4*	*1*.*00*	*1*.*6*	*5*.*9*
	*9–12*	*33*.*0*	*48*.*5*	*1*.*02*		
	*0–8*	*59*.*5*	*38*.*1*	*1*.*10*		
*MODEL 2*	***Maternal age (years)***					
	*≥20*	*79*.*0*	*83*.*2*	*1*.*00*	*0*.*5*	*2*.*3*
	*<20*	*21*.*0*	*16*.*8*	*1*.*13*		
	***Type of delivery***					
	*Vaginal/forceps*	*61*.*5*	*51*.*5*	*1*.*00*	*-4*.*5*	*1*.*0*
	*Cesarean*	*34*.*5*	*48*.*5*	*1*.*03*		
	***Maternal work status***					
	*Does not work outside home/on maternity leave*	*72*.*1*	*84*.*1*	*1*.*00*	*1*.*4*	*2*.*6*
	*Works outside home*	*27*.*9*	*15*.*9*	*1*.*13*		
*MODEL 3*	***Pacifier use***					
	*No*	*41*.*5*	*58*.*4*	*1*.*00*	*5*.*5*	*16*.*0*
	*Yes*	*58*.*5*	*41*.*6*	*1*.*40*		

^a^ Adjusted for sample weight of each municipality in each survey wave

^b^ Mean prevalence ratio obtained from the 1999 and 2008 survey waves in the hierarchical multilevel models

^c^ Obtained from generalized potential impact fraction calculation

^d^ Obtained from population attributable fraction calculation.

If pacifier use were eliminated, the expected decrease in EBF discontinuation (FAP) would be of 16 percentage points (Table 3). Higher maternal education, being an older mother and mother not working outside home/on maternity leave would lead to smaller proportional decreases in EBF when compared to pacifier use. However these effect sizes are also important to improve EBF prevalence. If all mothers delivered vaginally, only a slight decrease would be expected in EBF discontinuation (1%).

**Fig 3:**
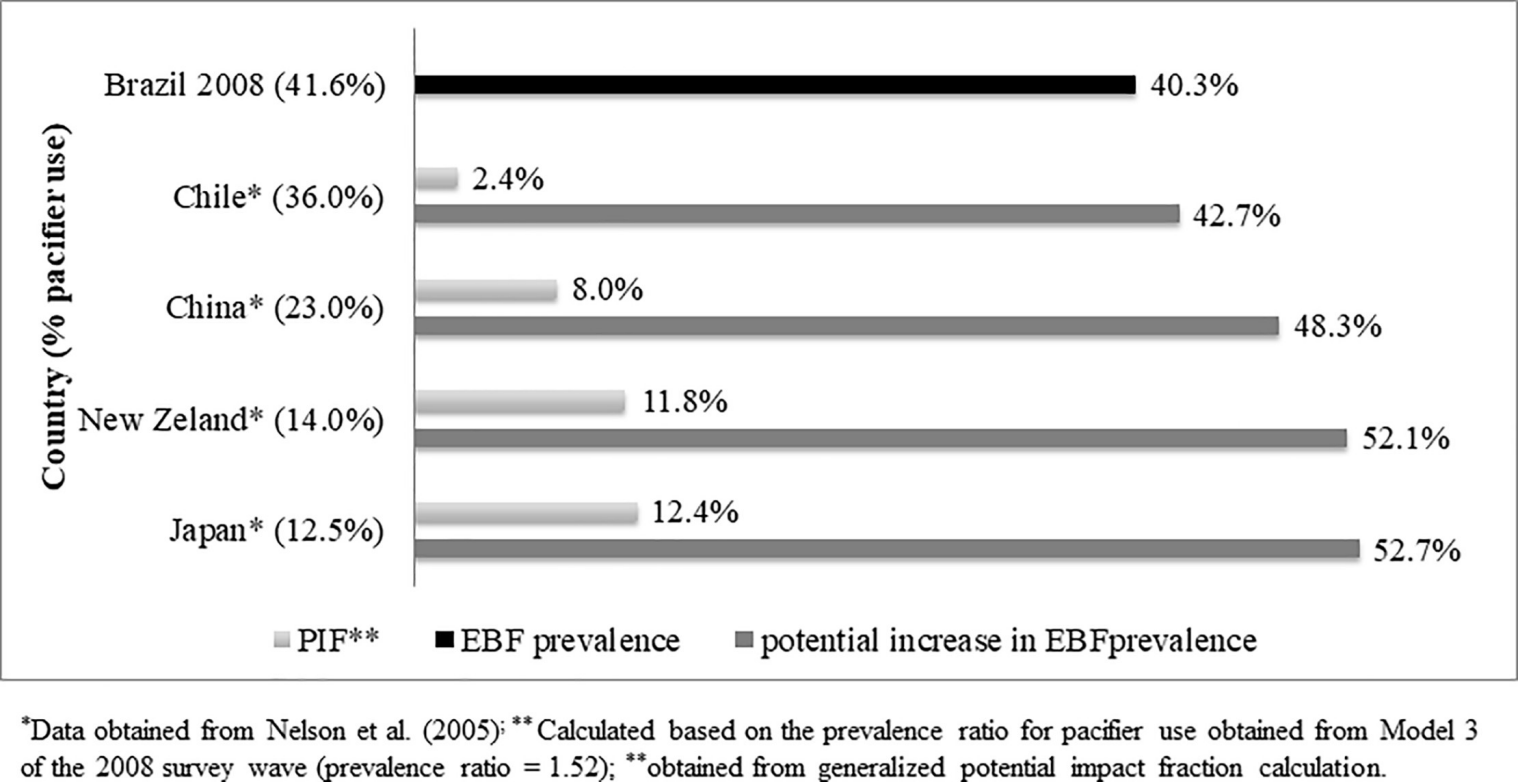
Expected additional decrease in exclusive breastfeeding discontinuation in simulated scenarios considering the Brazilian scenario with different frequencies of pacifier use.

If pacifier use decreased in Brazil from 41.6% (prevalence estimated at the latest survey in Brazil) to 14% (prevalence reported in New Zealand), there would be an expected additional decrease in EBF discontinuation of approximately 12 percentage points. This estimate assumes that all covariates assessed in this study would increase over time (Fig 3).

## Discussion

To our knowledge, this is the first study to quantify the contribution of pacifier use reduction to the increase in EBF prevalence observed in Brazil. The results suggest that a reduction in the prevalence of pacifier use may be an effective intervention to promote EBF.

EBF prevalence in Brazilian infants under 6 months increased from 3.1% in 1986 to 41.3% in 2008 [3]. The annual increment of 1.6 percentage point evidenced between 1999 and 2008 is higher than the increase reported in other countries [27]. During the period of study, multiple efforts to increase breastfeeding prevalence in multiple countries happened, including in Brazil. This included the implementation of the BFHI which used to strongly discourage the use of pacifiers in the maternity ward and currently counsels on advising women on the use and risks of pacifier [15, 28]. Based on the attributable potential impact fraction (PIF) of pacifier use reduction identified in this study we conclude that reducing pacifier use can indeed help accelerate the rate of improvement in EBF prevalence in Brazil [3, 27, 29].

Notwithstanding, despite the substantial decrease observed in pacifier use, this practice is still very common in Brazil [8]. Therefore we recommend for Brazil and other countries with similar contexts to consider adding recommendations on pacifier use in interventions for breastfeeding promotion at the population level [30–32].

Specifically, three types of public health strategies could be considered for pacifier use recommendations and interventions [33]. First, an ecological or population approach with general messages on promoting breastfeeding by avoiding pacifier use could be directed to the entire population [10, 34]. The second strategy, adopting a selective and individualized approach targeting groups at higher risk for pacifier use, the messages would require providing specific information on pros (e.g. SIDS risk reduction) and cons (e.g. interference with EBF) of pacifier use [35–38]. There is evidence that a reduction in pacifier use can improve breastfeeding outcomes, including a lower risk of EBF discontinuation, in high-risk populations, e.g., mothers working outside home [39], adolescent mothers and grandmothers [40]. Likewise, an individualized approach for pacifier use is needed among mothers at high risk for depression [41, 42].

Finally, a third strategy, also following an individually tailored or selective approach, would comprise messages directed to the mothers of infants who already use a pacifier, including information, for example, on limiting the period of pacifier use, restricting its use at critical moments such as when a baby may be more demanding (fussier) than usual, and, once the habit is established, providing support for stopping it in a gradual and timely manner [43]. Interventions following a selective approach among high-risk groups may be more cost-effective and advantageous if delivered in the context of peer counseling [44, 45], considering each situation individually [33], with emphasis on offering hands-on counseling and support for the management of breastfeeding difficulties [46, 47], adapting the message based on maternal and family perceptions with regard to pacifier use [48, 49], providing information on the pros and cons of pacifier use [42, 50–60], and strengthening the parents’ ability and confidence in soothing the baby [10, 43, 61]. We recommend that these strategies get tested using controlled study designs [8] and consider potential side effects such as the risk of SIDS [62] and the possibility that fussier babies would become more stressed if they do not receive a pacifier; before getting tested in large scale intervention study designs. However, these potential side effects need to be balanced against the potentially strong benefits that may result from improved breastfeeding outcomes resulting from reducing pacifier use as EBF has been found to be protective against SIDS and subsequent child psychosocial and emotional behavioral problems [63, 64].

Our study did not include populations living in rural areas, limiting the generalizability of our findings to rural areas. Nevertheless, our findings may be generalized to countries with a similarly high proportion of urban populations as Brazil including the rest of Latin America, and many European and Asian countries. Findings are also relevant for countries with a similar proportion or higher of pacifier use such as the one found in our study, including Italy [65], Denmark [66], Switzerland [28] and China [67]. Another limitation is the fact that data on age of introduction and intensity of pacifier use as well psychosocial factors that may influence the breastfeeding process including infant’s behavior (e.g., temperament and the mother’s breastfeeding intentions) were not collected. Due to its cross-sectional nature we cannot establish the temporal sequence of events between pacifier use and breastfeeding outcomes, thus reverse causality cannot be ruled out. In spite of these limitations, the fact that our findings are based on two national surveys that are representative of urban samples in a large country like Brazil and that both the regression analyses and the attributable potential impact fraction point to the same conclusions make our study a unique contribution to the literature.

### Conclusion

In conclusion, the knowledge about the attributable potential impact fraction of the decrease in pacifier use on the increase of EBF prevalence at a population level strengthens the case for issuing recommendations that address the pros and cons of pacifier use. Formative research on mother´s preference on pacifier use and EBF are needed to make such recommendations are effectively implemented across diverse populations.
